# Development of a broad-host-range *sacB*-based vector for unmarked allelic exchange

**DOI:** 10.1186/1756-0500-1-1

**Published:** 2008-02-26

**Authors:** Christopher J Marx

**Affiliations:** 1 Department of Organismic and Evolutionary Biology, Harvard University, 3083 Biological Laboratories, 16 Divinity Avenue, Cambridge, MA 02138, USA

## Abstract

**Background:**

Although genome sequences are available for an ever-increasing number of bacterial species, the availability of facile genetic tools for physiological analysis have generally lagged substantially behind traditional genetic models.

**Results:**

Here I describe the development of an improved, broad-host-range "in-out" allelic exchange vector, pCM433, which permits the generation of clean, marker-free genetic manipulations. Wild-type and mutant alleles were reciprocally exchanged at three loci in *Methylobacterium extorquens *AM1 in order to demonstrate the utility of pCM433.

**Conclusion:**

The broad-host-range vector for marker-free allelic exchange described here, pCM433, has the advantages of a high copy, general *Escherichia coli *replicon for easy cloning, an IncP *oriT *enabling conjugal transfer, an extensive set of restriction sites in its polylinker, three antibiotic markers, and *sacB *(encoding levansucrase) for negative selection upon sucrose plates. These traits should permit pCM433 to be broadly applied across many bacterial taxa for marker-free allelic exchange, which is particularly important if multiple manipulations or more subtle genetic manipulations such as point mutations are desired.

## Findings

### Background

The availability of genome sequences for hundreds of bacteria has helped reduce the disparity between the quality of physiological analyses available for these organisms and those possible for more traditional model organisms. Although genome-enabled approaches such as proteome and transcriptome analysis are now feasible for a great many systems, many of these non-traditional model organisms still have only limited genetic tools available for genetic manipulations. One particularly important tool is an allelic exchange vector to perform reverse genetic analysis. This generally involves homologous recombination of a selectable marker, such as an antibiotic resistance cassette, into the desired site on the chromosome. Issues arise, however, when the planned number of genetic manipulations outnumbers the number of markers available, or if the effect of mutations such as nucleotide substitutions, is desired.

Generating more genetic multiple manipulations than the number of markers requires a more sophisticated approach than just homologous recombination. One strategy used successfully in *Escherichia coli *is to use a combination of *in vivo *restriction of a donor plasmid with a transiently-expressed, highly active recombination system based on Lambda Red recombinase [1]. The goal here is to perform recombination in the complete absence of selection at a frequency sufficiently high (>1%) to be screened for directly. Although this is a rather powerful system, it is unclear whether Lambda Red can be utilized outside of some taxa within the γ-proteobacteria. Attempts to introduce antibiotic markers into chromosomal loci the α-proteobacterium used in this work, *Methylobacterium extorquens *AM1, failed to produce any desired recombinants (Marx, unpublished). A second option for generating multiple mutations is to excise the markers utilized with an *in vivo*, site-specific recombinase system. Options that have been employed to this end include the recombinase/recognition site pairs *cre/lox *[2] and Flp/*FRT *[3]. Although broad-host-range systems like this have been developed (for example [4]), there remain some disadvantages. Notably, although the selectable marker itself is removed, recombination between the two, collinear recombinase recognition sites leaves behind a single copy of this site. Depending on the system in use, the minimal scar left behind is the recombination recognition sites of 34 (*loxP*) and 48 (*FRT*) bp in length. Additionally, multiple cloning sites between the two regions of homology cloned into the donor vector will also be left behind in the scar after marker removal. The introduction of scars is a particular problem when trying to cleanly assess the physiological effect of mutations that are more 'subtle' than knockouts or insertions, for example, a point mutation that results in an amino acid substitution in the encoded protein.

**Figure 1:**
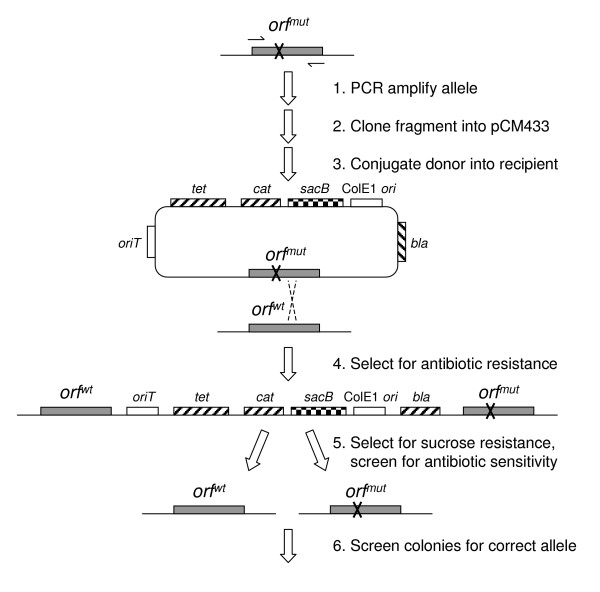
**Strategy for pCM433-based "in-out" allelic exchange**. Scheme depicted for the example of introducing a mutant allele with a point mutation (*orf*^*mut*^) in place of the wt allele (*orf*^*wt*^). PCR amplification of *orf*^*mut *^with primers complementary to the boundaries of the coding sequence, followed by cloning of the resulting fragment into pCM433, results in the desired *orf*^*mut *^donor plasmid. This donor can then be conjugated into the *orf*^*wt *^recipient strain, for which selection of one of the three encoded antibiotic resistances (Ap, Cm, Tc) will result in strains that have experienced recombination at the desired allele, and incorporation of the donor plasmid into the chromosomal locus. Although the recombination is drawn here to have occurred to the right of the point mutation, resulting in the *orf*^*wt *^upstream, recombination to the left of the point mutation would situate *orf*^*mut *^upstream. Selection for sucrose resistance, and screening for antibiotic sensitivity results in clean exchange of the *orf*^*mut *^allele or reversion to *orf*^*wt*^. Finally, isolates bearing clean, single copies of the locus can be screened to identify a strain bearing the desired allele.

A third option for multiple genetic manipulations, which also avoids leaving behind undesired scars, is to use an "in-out" system (Figure 1[5]). The basic idea behind these techniques is to first employ positive selection to select for single crossover integration of the entire donor vector due to recombination between a cloned region spanning the desired mutation in the vector and the corresponding chromosomal site. In the second step, negative selection is used to select for isolates that have recombined out the vector sequence. If the second recombination event excising the vector occurs on the same side of the introduced mutation as the first recombination event that introduced it onto the chromosome, the original chromosomal locus will be restored unchanged. If the second recombination event occurs on the opposite side of the introduced mutation, however, this results in excision of the original allele and the new mutation remains. As such, negative selection results in colonies with both resulting final states, as well as some percentage of false-positives that are resistant but have not excised the vector. As long as the false positives do not dominate, and the recombination rates to each side of the introduced mutation are reasonably balanced, screening of a modest collection of resulting recombinants will generate the desired unmarked mutation.

An "in-out" allelic exchange vector for generating unmarked mutations therefore must be able to be introduced into the recipient organism, be incapable of vegetative replication, and bear appropriate markers for positive and negative selection. Positive selection is generally accomplished using any number of antibiotic resistance genes, whereas comparably fewer options for negative selection generally exist. The most commonly used techniques are to use streptomycin (Sm) sensitivity, which comes as a pleiotropic effect of expressing the tetracycline (Tc) efflux pump [6], or sucrose-sensitivity that results from expression of levansucrase, encoded by *sacB *[7]. Levansucrase activity is lethal in the presence of sucrose for most gram-negative bacteria. This paper presents a facile, broad-host-range "in-out" system based on *sacB *that has been specifically designed to allow facile unmarked allelic exchange in a wide variety of bacterial taxa. In order to test this system, allelic exchange has been performed at three different loci in *M. extorquens *AM1 [8,9].

## Results and Discussion

### Construction of the "in-out" allelic exchange vector pCM433

**Figure 2:**
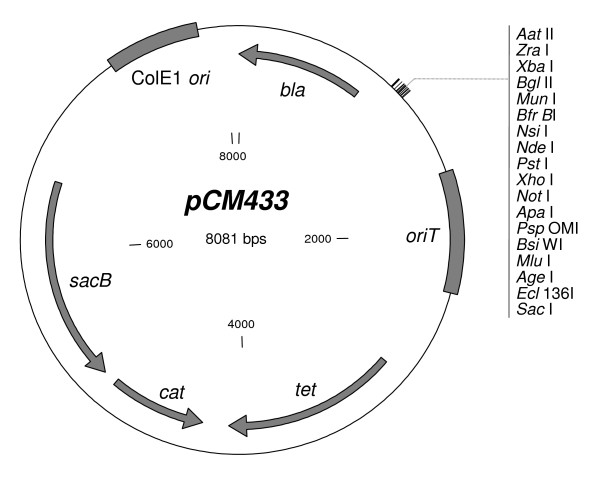
**Broad-host-range allelic exchange vector pCM433**. Plasmid map of pCM433 [GenBank:EU118176] indicating the available restriction sites in the multiple-cloning site, as well as key plasmid features: *bla *(encodes betalactamase; Ap^R^), *oriT *(IncP origin of conjugal transfer), *tet *(encodes Tc efflux pump), *cat *(encodes Cm actetyltransferase), *sacB *(levansucrase; confers sucrose sensitivity), and ColE1 *ori *(high copy *E. coli *origin of replication). Plasmid map was generated using Clone Manager 7 (Sci Ed Central).

In order to generate a facile system for marker-free allelic exchange across a wide variety of bacterial species, the *loxP*-flanked kanamycin (Km) resistance cassette of the broad-host-range marker-recycling vector, pCM184 [4] was first excised and replaced with a synthetic linker that introduced three new restriction sites to the extensive multiple-cloning sites. Subsequently, a fragment from pDS132 [10] bearing *sacB *and *cat *(encoding levansucrase and chloramphenicol (Cm) acetyltransferase, respectively) was introduced, generating pCM433 (Figure 2). It may be noted that initial attempts were made to take advantage of the potential negative selection (Sm sensitivity) afforded by expression of the Tc efflux pump present on pCM184. Sm sensitivity was found to be enhanced in *tet *bearing cells, but the sensitivity was too modest to be utilized effectively for negative selection (Marx, unpublished results).

### Allelic exchange at three loci in *M. extorquens *AM1

Three loci of interest in *M. extorquens *AM1 were chosen to test the utility of pCM433 for allelic exchange. These loci were *hprA *(encodes hydroxypyruvate reductase, a key enzyme of the serine cycle for assimilation of formaldehyde into biomass, [11]), *mptG *(encodes β-ribofuranosylaminobenzene 5'-phosphate synthase, the first dedicated enzyme for the synthesis of tetrahydromethanopterin, the C_1_-carrier molecule used for this organism's formaldehyde oxidation pathway [12,13]), and *crtI *(encodes phytoene desaturase, a necessary enzyme for carotenoid biosynthesis [14]).

In all cases, constructs were made to convert the allele from wild-type (wt) to mutant, and the reciprocal reversion of mutant to wt. To accomplish this, both the ancestral, wt allele and the deletion (Δ*hprA*, *ΔmptG*) or insertion (*crtI*^502^, generated by insertion of IS*phoA/hah-*Tc into *crtI*, followed by Cre-mediated excision of all but 132 bp of the IS [14]) alleles were amplified by PCR, cloned into pCR2.1, sequenced, and then introduced into pCM433. Each of these donor plasmids were then introduced into the appropriate target strain via triparental conjugations and plated onto Tc plates (also containing Rif for counter-selection against *E. coli*). Tc^R ^transconjugants were obtained at a frequency of 10^-6 ^to 10^-7^. In some cases, even these single-crossover recombinants that contained both the wild-type and mutant alleles exhibited a phenotype. For example, the pool of single-crossover intermediates from either pCM441 (wt *crtI *allele) inserted into the white CM502 strain or pCM440 (defective *crtI*^502 ^allele) inserted into the pink CM501 strain each contained Tc^R ^colonies of both colors. As such, one pink and one white isolate from the conjugation into each background were isolated (CM1263 (white) and CM1264 (pink) from CM502, and CM1265 (pink) and CM1266 (white) from CM501). A polar effect of pCM433 insertion into this site was clearly observed. Irrespective of whether the wt allele was being introduced into the mutant, or vise versa, strains with the wild-type allele upstream, proximal to the gene's promoter (as determined by PCR analysis for strains CM1264 and CM1265), were pink, carotenoid-containing colonies, whereas the other strains (CM1263 and CM1266) had the *crtI*^502 ^allele upstream of pCM433 and were white.

In order to select for recombinants that have excised the vector, suspensions of Tc^R ^isolates were diluted and plated onto plates containing various levels of sucrose (2.5, 5, and 10% w/v). At all sucrose levels sucrose-resistant colonies were obtained at a frequency of 10^-4 ^to 10^-5^. These colonies were then screened for Tc sensitivity (indicating the expected loss of the pCM433-based construct), as well as the expected mutant phenotype (inability to grow on methanol for *ΔhprA *and *ΔmptG*, white colonies (versus pink) for *crtI*^502^). These were confirmed via PCR analysis using primers situated outside the region of the locus where recombination occurred. In the cases presented here, differences in the size of amplified products sufficed to distinguish the alleles used, but primers designed to distinguish single-nucleotide substitutions (or sequencing) have been used in subsequent studies (Chou and Marx, unpublished). Overall, a false positive rate of sucrose^R^, Tc^R ^strains generated here in *M. extorquens *AM1 was 26% (105/402). It should be noted, however, that the range of frequencies varied from 0% to 78% for different construct/recipient pairs. This is likely related to the rate of recombination for the flanking regions of each locus as compared to the rate of generating sucrose-resistance from other mechanisms. For all three loci, wild-type alleles were replaced by mutant alleles, and vise versa. In subsequent work, dozens of allelic exchanges including the introduction of single-nucleotide substitutions have been successfully performed utilizing this system (Chou and Marx, unpublished).

## Conclusion

The broad-host-range vector for marker-free allelic exchange described here has several features that greatly facilitate its use in various systems. First, unlike a number of similar vectors, such as pDS132 from which much some of the construct derives [10], pCM433 relies upon a pUC-derived ColE1 replicon, such that it can be maintained and easily purified in high quantities (5–10 μg DNA from 1.5 ml liquid culture) in any desired *E. coli *strain. Second, pCM433 contains a polylinker containing a substantially larger number of restriction sites than comparable tools we are aware of [3,10], facilitating the introduction of cloned DNA fragments. Third, the presence of three antibiotic markers on pCM433 permits use in a wide range of organisms in which they are applicable. Finally, pCM433 maintains features typically found in other broad-host-range systems such as the presence of an IncP *oriT *that allows conjugation to be utilized for delivery into the recipient strain.

Limitations for the use of this allelic exchange system are that it will not work in enterics where the ColE1 replicon is maintained vegetatively, and negative selection by using sucrose to select against cells expressing *sacB *must be effective. In this regard, the observed false positive rate observed in *M. extorquens *AM1 of 26% was sufficiently low to have allowed the facile exchange of alleles at three different loci, all of which generated the expected changes in phenotype. Potential causes for the variability in the false positive rate observed with some constructs include differences in the relative rates of homologous recombination between the transiently duplicated genomic locus, or local differences in rates of *sacB *mutation (point mutations or transposition) that result in Suc^R ^without the expected homologous recombination. Another consideration that should be kept in mind with "in-out" systems is that the intermediate genotype created by a single-crossover event between the fragment cloned into pCM433 and the chromosomal locus must be viable (see Figure 1). Because of this, it may be useful to clone intact gene products where lethality of resulting strains is expected, but this alone will not necessarily prevent potential polar effects. As long as at least one of the two potential intermediate states is viable, however, removal of the entire vector sequence during the second recombination event (selected for by sucrose) results in single-copy, unmarked loci, removing any concerns of polarity. This marker-free system should therefore permit allelic exchange in a wide variety of organisms, many of which otherwise lack the well-developed genetic systems of more intensively-studied model systems. This will be particularly important for studying the phenotypic effect of more subtle genetic manipulations such as point mutations.

## Methods

### Media, growth conditions, and genetic techniques

*M. extorquens *AM1 strains were grown at 30°C on agar plates with "Hypho" minimal salts medium [15]; *E. coli *were grown at 37°C on Luria-Bertani agar [16]. Substrates and antibiotics were used at the following concentrations: methanol (125 mM), succinate (15 mM), sucrose (5% w/v unless otherwise stated), 50 μg/ml Ap (ampicillin), 20 μg/ml Cm, 50 μg/ml Km, 50 μg/ml Rif (rifamycin), 35 μg/ml Sm, and 10 μg/ml Tc.

Tri-parental conjugations were performed by mixing the *E. coli *strain with the donor plasmid, the *M. extorquens *AM1 recipient strain, and an *E. coli *strain with the helper plasmid pRK2073 [17]. This mixture was grown overnight on permissive Nutrient agar [16] plates at 30°C before introducing some of the mix (either by streaking with a loop or by washing with Hypho and re-plating) onto selective medium containing an appropriate C source, Rif for counter-selection against *E. coli *[9], and the selective antibiotic (Tc for pCM433-based donors; neither Ap nor Cm works effectively in *M. extorquens *AM1, Marx, unpublished). Sucrose selection was accomplished by suspending a loop of a given strain in 100 μl Hypho (approximately 10^9 ^ml^-1^) and plating 50 μl of a 10^-2 ^dilution of this suspension onto Hypho plates containing an appropriate C source (generally succinate) and 5% sucrose. Resulting strains were tested for Tc sensitivity, additional expected phenotypes (depending on the locus and allele being exchanged), and additionally, the chromosomal organization of all strains constructed was confirmed through PCR analysis. DNA concentrations were determined using a ND-1000 spectrophotometer (NanoDrop).

### Construction of plasmids and generation of strains

In order to generate the allelic exchange vector pCM433, the Km resistance cassette of pCM184 [4] was excised with *Nde*I and *Sac*II, and the remaining 5.4 kb vector backbone was ligated together with a synthetic linker designed to introduce three additional, unique cloning sites into the final vector (*Pst*I, *Xho*I, and *Not*I). The linker was formed by boiling, and then slowly re-annealing at room temperature, a mixture of two oligos, CM-link1f (tatgctgcagctcgagcggccgc) and CM-link1r (ggccgctcgagctgcagca), which were designed to have complementary overhangs to *Nde*I and *Sac*II. The resulting plasmid, pCM432, was then transformed into the *dam dcm E. coli *strain, C2925H (*ara-14 leuB6 fhuA31 lacY1 tsx78 glnV44 galK2 galT22 mcrA dcm-6 hisG4 rfbD1 R(zgb210::Tn10) Tc*^*S*^*endA1 rspL136 (Sm*^*R*^*) dam13::Tn9 (Cm*^*R*^*) xylA-5 mtl-1 thi-1 mcrB1 hsdR2*, New England Biolabs), enabling digestion at an otherwise methylated, and therefore blocked, *Msc*I site. The 2.7 kb *Xba*I-*Xma*I fragment of pDS132 [10] containing *sacB *and *cat *was then purified, blunted with Klenow enzyme, and ligated with the *Msc*I-digested pCM432 vector to generate pCM433 (see Figure 2). A construct with the *sacB-cat *fragment in the opposite orientation, pCM433r, was also obtained. All plasmids and strains used are referenced in Table 1.

**Table 1:** *Methylobacterium *Strains and Plasmids Used in This Study

**Strain/Plasmid**	**Relevant Properties**	**Reference**
Strains*		
AM1-W	*crtI::*IS*phoA/hah *(i.e. *crtI*^502^) strain; white	[14]
C2925H	Cm^R^, Sm^R^;	New England Biolabs
CM253.1	Δ*mptG*	[18]
CM501	Isolate of wild-type *M. extorquens *AM1; pink	This study
CM502	Isolate of AM1-W; white "wild-type" with *crtI*^502 ^allele	This study
CM508	Isolate of Δ*mptG *strain CM253.1	This study
CM1122	Km^R^; Δ*hprA::kan*; pCM431 integrated into CM501	This study
CM1123	Km^R^; Δ*hprA::kan crtI*^502^; pCM431 integrated into CM502	This study
CM1203	Δ*hprA*; *kan *deleted from CM1122	This study
CM1204	Δ*hprA crtI*^502^; *kan *deleted from CM1123	This study
CM1263	Tc^R^, sucrose^S^; pCM441 integrated into CM501; *crtI*^502 ^upstream; white	This study
CM1264	Tc^R^, sucrose^S^; pCM441 integrated into CM501; *crtI *upstream; pink	This study
CM1265	Tc^R^, sucrose^S^; pCM440 integrated into CM502; *crtI *upstream; pink	This study
CM1266	Tc^R^, sucrose^S^; pCM440 integrated into CM502; *crtI*^502 ^upstream; white	This study
*M. extorquens *AM1	Rif^R ^isolate; pink	[9]
**Plasmids**		
pCM157	Tc^R^; broad-host-range *cre *expression vector	[4]
pCM184	Ap^R^, Km^R^, Tc^R^; broad-host range *cre-lox *allelic exchange vector	[4]
pCM411	Ap^R^, Km^R^; *mptG *region cloned into pCR2.1	This study
pCM417	Ap^R^, Km^R^; *crtI *region cloned into pCR2.1	This study
pCM424	Ap^R^, Km^R^; Δ*mptG *region cloned into pCR2.1	This study
pCM426	Ap^R^, Km^R^; *crtI*^502 ^region cloned into pCR2.1	This study
pCM428	Ap^R^, Km^R^; pCR2.1 with *hprA *upstream flank	This study
pCM429	Ap^R^, Km^R^; pCR2.1 with *hprA *downstream flank	This study
pCM430	Ap^R^, Km^R^, Tc^R^; pCM184 with *hprA *upstream flank from pCM428	This study
pCM431	Ap^R^, Km^R^, Tc^R^; pCM430 with *hprA *downstream flank from pCM429	This study
pCM432	Ap^R^, Tc^R^; synthetic linker introduced into pCM184	This study
pCM433	Ap^R^, Cm^R^, Tc^R^; pCM432 with *sacB-cat *fragment from pDS132; broad-host-range *sacB*-based allelic exchange vector	This study
pCM433r	Ap^R^, Cm^R^, Tc^R^; pCM433 with *sacB-cat *fragment in the opposite orientation as in pCM433	This study
pCM434	Ap^R^, Km^R^; *hprA *region cloned into pCR2.1	This study
pCM435	Ap^R^, Cm^R^, Tc^R^; *hprA *region from pCM434 cloned into pCM433	This study
pCM436	Ap^R^, Cm^R^, Tc^R^; *mptG *region from pCM411 cloned into pCM433	This study
pCM437	Ap^R^, Cm^R^, Tc^R^; Δ*mptG *region from pCM424 cloned into pCM433	This study
pCM438	Ap^R^, Km^R^; Δ*hprA *region cloned into pCR2.1	This study
pCM439	Ap^R^, Cm^R^, Tc^R^; Δ*hprA *region from pCM438 cloned into pCM433	This study
pCM440	Ap^R^, Cm^R^, Tc^R^; *crtI *region from pCM417 cloned into pCM433	This study
pCM441	Ap^R^, Cm^R^, Tc^R^; *crtI*^502 ^region from pCM426 cloned into pCM433	This study
pCR2.1	Ap^R^, Km^R^; PCR cloning vector	Invitrogen
pDS132	Ap^R^, Cm^R^;, *sacB*-based allelic exchange vector with *λpir*-dependent R6K*ori*	[10]
pRK2073	Sm^R^; helper plasmid supplying IncP *tra *functions	[17]

*All *M. extorquens *AM1 strains are also Rif^R ^[9]. Antibiotic resistances are indicated as follows, Ap (ampicillin), Cm (chloramphenicol), Km (kanamycin), Rif (rifamycin), Sm (streptomycin), Tc (tetracycline).

A series of constructs and strains were generated in order to test the ability of pCM433 to enable unmarked allelic exchange at three distinct loci in the *M. extorquens *AM1 chromosome. Donor constructs for allelic exchange at the *mptG *locus were generated by first amplifying a region including *mptG *from CM501 (an isolate of wild-type, Rif^R ^*M. extorquens *AM1 [9]), or the corresponding region from the *ΔmptG *strain, CM508 (an isolate of CM253.1 [18]), each of which were ligated into pCR2.1 (Invitrogen) to generate pCM411 and pCM424, respectively. These PCR-amplified inserts (and all other alleles described below that were cloned into pCR2.1) were sequenced to confirm no PCR errors were introduced during amplification. The 2.1 kb *Apa*I-*Bam*HI fragment of pCM411 containing the *mptG *region was then introduced into pCM433 that had been digested with *Apa*I and *Bgl*II, resulting in the donor vector pCM436. Similarly, the 1.3 kb *Sac*I-*Xho*I fragment of pCM438 with the *ΔhprA *region was cloned into the same sites of pCM433 to generate the donor vector pCM439. This allowed the use of pCM436 (containing the wild-type *mptG *allele) to reverse the lesion found in CM508, while pCM437 (*ΔmptG *allele) was introduced into CM501 to do the opposite, generating the deletion in a single step.

Similarly, donor constructs for allelic exchange at the *crtI *locus were generated by first amplifying a region including *crtI *(encodes phytoene desaturase) from the pink CM501 strain, or the corresponding region from the white *crtI::*IS*phoA/hah *(i.e. *crtI*^502^) strain, CM502 (an isolate of AM1-W [14]). These fragments were ligated into pCR2.1 (Invitrogen) to generate pCM417 and pCM426, respectively. The 1.6 kb *Bam*HI-*Nsi*I fragment of pCM411 containing the *crtI *region was then introduced into pCM433 that had been digested with *Bgl*II and *Nsi*I, resulting in the donor vector pCM440. Similarly, the 1.7 kb *Bam*HI-*Not*I fragment of pCM426 with the *crtI*^502 ^region was cloned between the *Bgl*II and *Not*I sites of pCM433 to generate the donor vector pCM441. This allowed the use of pCM440 (containing the wild-type *crtI *allele) to reverse the lesion found in CM502, while pCM441 (*crtI*^502 ^allele) was introduced into CM501 to do the opposite, generating the insertion allele.

Finally, for the third locus, *hprA*, an antibiotic-resistance free deletion strain was generated initially using a previously developed *cre-lox *system [4]. In contrast to the system described here using pCM433, the process to generate the *ΔhprA *strain was substantially more involved (and resulted in leaving behind a *loxP *scar). First, the regions upstream and downstream of *hprA*, were amplified separately and cloned into pCR2.1 (Invitrogen) to generate pCM428 and pCM429, respectively. The 0.5 kb upstream region was then excised from pCM428 using *Bgl*II and *Not*I and ligated into the same sites of pCM184 to generate pCM430. Into this plasmid, the 0.6 kb *Apa*I-*Sac*I fragment from pCM429 was cloned into the same sites to generate the donor plasmid pCM431. As previously described [4], this plasmid was introduced into both the wild-type (pink) *M. extorquens *AM1 strain, CM501, as well as the otherwise isogenic white strain with a *crtI*^502 ^allele, CM502, leading to the isolation of the *hprA::kan *strains CM1122 and CM1123, respectively. pCM157 (expressing Cre recombinase) was introduced into these two strains to catalyze the excision of the *kan *cassette, and was subsequently cured, ultimately resulting in the antibiotic-resistance free *ΔhprA *strains CM1203 and CM1204 used below.

Donor constructs for allelic exchange of the *hprA *locus were generated by first amplifying a region including *hprA *from CM501, or the corresponding region from the *ΔhprA *strain generated above, CM1203. Ligation of these fragments into pCR2.1 (Invitrogen) generated pCM434 and pCM438, respectively. The 2.2 kb *Apa*I-*Bam*HI fragment of pCM434 containing the *hprA *region was introduced into pCM433 that had been digested with *Apa*I and *Bgl*II, resulting in the donor vector pCM434. Similarly, the 1.3 kb *Spe*I-*Nsi*I fragment of pCM438 with the *ΔhprA *region was cloned between the *Xba*I and *Nsi*I sites of pCM433 to generate the donor vector pCM439. This allowed the use of pCM434 (containing the wild-type *hprA *allele) to reverse the lesion found in CM1203, while pCM439 (Δ*hprA *allele) was introduced into CM501 to do the opposite, generating the deletion in a single step.

## Availability and requirements

### Nucleotide sequence accession number and plasmid database information

The sequence of pCM433 has been deposited [GenBank:EU118176] and the plasmid has been deposited with Addgene (, Plasmid 15670).

## Authors' contributions

CJM designed and carried out all experiments and drafted the final manuscript.

## References

[B1] Herring CD, Glasner JD, Blattner FR (2003). Gene replacement without selection: regulated suppression of amber mutations in *Escherichia coli*. Gene.

[B2] Ayres EK, Thomson VJ, Merino G, Balderes D, Figurski DH (1993). Precise deletions in large bacterial genomes by vector-mediated excision (VEX). The *trfA *gene of promiscuous plasmid RK2 is essential for replication in several gram-negative hosts. J Mol Biol.

[B3] Hoang TT, Karkhoff-Schweizer RR, Kutchma AJ, Schweizer HP (1998). A broad-host-range *Flp*-FRT recombination system for site-specific excision of chromosomally-located DNA sequences: application for isolation of unmarked *Pseudomonas aeruginosa* mutants. Gene.

[B4] Marx CJ, Lidstrom ME (2002). Broad-host-range *cre-lox* system for antibiotic marker recycling in gram-negative bacteria. BioTechniques.

[B5] Hamilton CM, Aldea M, Washburn BK, Babitzke P, Kushner SR (1989). New method for generating deletions and gene replacements in *Escherichia coli*. J Bacteriol.

[B6] Maloy SR, Nunn WD (1981). Selection for loss of tetracycline resistance by *Escherichia coli*. J Bacteriol.

[B7] Gay P, Le Coq D, Steinmetz M, Ferrari E, Hoch JA (1983). Cloning structural gene *sacB*, which codes for exoenzyme levansucrase of *Bacillus subtilis*: expression of the gene in *Escherichia coli*. J Bacteriol.

[B8] Peel D, Quayle JR (1961). Microbial growth on C1 compounds: 1. Isolation and characterization of *Pseudomonas* AM1. Biochem J.

[B9] Nunn DN, Lidstrom ME (1986). Isolation and complementation analysis of 10 methanol oxidation mutant classes and identification of the methanol dehydrogenase structural gene of *Methylobacterium* sp. strain AM1. J Bacteriol.

[B10] Philippe N, Alcaraz JP, Coursange E, Geiselmann J, Schneider D (2004). Improvement of pCVD442, a suicide plasmid for gene allele exchange in bacteria. Plasmid.

[B11] Chistoserdova LV, Lidstrom ME (1992). Cloning, mutagenesis, and physiological effect of a hydroxypyruvate reductase gene from *Methylobacterium extorquens* AM1. J Bacteriol.

[B12] Chistoserdova L, Vorholt JA, Thauer RK, Lidstrom ME (1998). C1 transfer enzymes and coenzymes linking methylotrophic bacteria and methanogenic Archaea. Science.

[B13] Scott JW, Rasche ME (2002). Purification, overproduction, and partial characterization of beta-RFAP synthase, a key enzyme in the methanopterin biosynthesis pathway. J Bacteriol.

[B14] Van Dien SJ, Marx CJ, O'Brien BN, Lidstrom ME (2003). Genetic characterization of the carotenoid biosynthetic pathway in *Methylobacterium extorquens* AM1 and isolation of a colorless mutant. Appl Environ Microbiol.

[B15] Attwood MM, Harder W (1972). A rapid and specific enrichment procedure for *Hyphomicrobium* spp. Antonie Van Leeuwenhoek.

[B16] Sambrook J, Fritsch EF, Maniatis T (1989). Molecular Cloning: a Laboratory Manual.

[B17] Figurski DH, Helinski DR (1979). Replication of an origin-containing derivative of plasmid RK2 dependent on a plasmid function provided *in trans*. Proc Natl Acad Sci USA.

[B18] Marx CJ, Chistoserdova L, Lidstrom ME (2003). Formaldehyde-detoxifying role of the tetrahydromethanopterin-linked pathway in *Methylobacterium extorquens* AM1. J Bacteriol.

